# Validation of a Smartphone App for the Assessment of Sedentary and Active Behaviors

**DOI:** 10.2196/mhealth.6974

**Published:** 2017-08-09

**Authors:** Meynard John Toledo, Eric Hekler, Kevin Hollingshead, Dana Epstein, Matthew Buman

**Affiliations:** ^1^ Arizona State University School of Nutrition and Health Promotion Phoenix, AZ United States; ^2^ Phoenix Veterans Affairs Health Care System Phoenix, AZ United States; ^3^ Arizona State University College of Nursing and Health Innovation Phoenix, AZ United States

**Keywords:** Sedentary and physical activity measurement, smartphone daily-log app, self-monitoring app, BeWell24

## Abstract

**Background:**

Although current technological advancements have allowed for objective measurements of sedentary behavior via accelerometers, these devices do not provide the contextual information needed to identify targets for behavioral interventions and generate public health guidelines to reduce sedentary behavior. Thus, self-reports still remain an important method of measurement for physical activity and sedentary behaviors.

**Objective:**

This study evaluated the reliability, validity, and sensitivity to change of a smartphone app in assessing sitting, light-intensity physical activity (LPA), and moderate-vigorous physical activity (MVPA).

**Methods:**

Adults (N=28; 49.0 years old, standard deviation [SD] 8.9; 85% men; 73% Caucasian; body mass index=35.0, SD 8.3 kg/m2) reported their sitting, LPA, and MVPA over an 11-week behavioral intervention. During three separate 7-day periods, participants wore the activPAL3c accelerometer/inclinometer as a criterion measure. Intraclass correlation (ICC; 95% CI) and bias estimates (mean difference [δ] and root of mean square error [RMSE]) were used to compare app-based reported behaviors to measured sitting time (lying/seated position), LPA (standing or stepping at <100 steps/minute), and MVPA (stepping at >100 steps/minute).

**Results:**

Test-retest results suggested moderate agreement with the criterion for sedentary time, LPA, and MVPA (ICC=0.65 [0.43-0.82], 0.67 [0.44-0.83] and 0.69 [0.48-0.84], respectively). The agreement between the two measures was poor (ICC=0.05-0.40). The app underestimated sedentary time (δ=-45.9 [-67.6, -24.2] minutes/day, RMSE=201.6) and overestimated LPA and MVPA (δ=18.8 [-1.30 to 38.9] minutes/day, RMSE=183; and δ=29.3 [25.3 to 33.2] minutes/day, RMSE=71.6, respectively). The app underestimated change in time spent during LPA and MVPA but overestimated change in sedentary time. Both measures showed similar directions in changed scores on sedentary time and LPA.

**Conclusions:**

Despite its inaccuracy, the app may be useful as a self-monitoring tool in the context of a behavioral intervention. Future research may help to clarify reasons for under- or over-reporting of behaviors.

## Introduction

Lack of physical activity (PA; <150 minutes of moderate-vigorous PA per week [1]) and sedentary behavior (ie, sitting/reclining with low energy expenditure while awake [2-4]) are associated with increased risks for cardiometabolic diseases, such as hypertension, dyslipidemia, diabetes, and coronary heart disease. As such, accurate assessment of these behaviors is integral in developing potent behavioral interventions aimed at improving overall cardiometabolic health.

Assessment of PA and sedentary behaviors have improved significantly in recent years. Technological advancements have led to the development of sensors that can objectively and accurately measure these behaviors [5-7]. Although these sensors have led to an unprecedented objectivity in PA measurement, the self-report method is still ubiquitous in PA research; this is because it offers advantages over objective methods [8]. Accelerometers do not capture the contextual information associated with these activities. Researchers studying specific domains of these behaviors (ie, leisure-time, occupational, or transportation) still rely on self-reports to isolate the behaviors that occur in each of these domains. Distinguishing which domains these behaviors occur in is necessary for developing and evaluating targeted interventions to modify these domain-specific behaviors [9]. Thus, self-reports remain an important method of measurement for PA and sedentary behaviors.

Various types of PA questionnaires have been developed, ranging from global questionnaires to detailed quantitative histories. Strath et al [8] classified PA questionnaires into three broad categories: global, short recalls, and quantitative history. Global PA questionnaires are usually short (2 to 4 items) and provide an overview of an individual’s overall activity level. These questionnaires are primarily used to identify whether individuals meet the PA standard, or classify individuals according to their PA levels (eg, active vs inactive). In contrast, short recalls provide a measure of an individual’s PA level, as classified by the dimension of intensity level or domain. Quantitative history questionnaires are detailed measures that are used to understand the types and intensity of PAs that contribute to mortality or morbidity. A systematic review of studies that evaluated the reliability and objective criterion-related validity of new and existing PA questionnaires examined 65 studies that looked at a total of 96 PA questionnaires [10]. The results revealed poor-to-moderate validity, with median validity coefficients ranging from 0.30-0.39 for existing (and from 0.25-0.41 for new) PA questionnaires. Furthermore, other studies have shown that although these questionnaires show acceptable agreement for structured vigorous-intensity PAs, they are less accurate for more prevalent lower-intensity activities [11-14]. Similar patterns of accuracy and reliability are also consistent with existing instruments that measure sedentary behavior. For example, habitual domain-specific sedentary behaviors tend to have higher correlations with criterion measures than overall sedentary time (0.14-0.83 vs 0.07-0.61) [15]. This pattern is mainly due to the high cognitive demands associated with reporting usual daily activities [14].

The PA diary is another type of self-report measure of PA and sedentary behavior that is often used to obtain a detailed hour-by-hour or activity-by-activity record [8]. An example of a daily diary is the Bouchard Physical Activity Record, which asks participants to categorize their activity every 15 minutes in 1 of 9 types of movement behaviors [16]. Using a PA diary is advantageous because it includes detailed information on dimensions and domains of PA and sedentary behavior, and is less subject to memory bias than other methods. However, the detail of information and frequency of reporting required causes significant participant burden, which limits its use in long-term studies. A possible solution to this problem is to leverage mobile technology as a platform to deliver these PA and sedentary behavior measurement tools. The ubiquitous use of mobile phones offers an opportunity to capture self-report behaviors in real-time and with minimal recall bias [17]. Utilizing mobile platforms to deliver these questionnaires can lead to an easy-to-use and a readily accessible version of PA and sedentary behavior assessment tool, which could significantly lessen the burden associated with traditional diaries.

The purpose of this paper is to evaluate the reliability, validity, and sensitivity to change of a smartphone-based app (*BeWell24*), which is designed to assess sedentary behavior, light-intensity physical activity (LPA), and moderate-vigorous physical activity (MVPA), as compared to an objective measure (activPAL3c; PAL Technologies Ltd, Scotland, UK). Evaluating the accuracy of the app is the first step in establishing its usefulness as a self-report tool to measure PA and sedentary behavior in free-living environments, and provide rationale for future studies to evaluate its capacity in measuring context-specific activities.

## Methods

### Recruitment

Participants were US veterans that were receiving care at a regional Veterans Health Administration (VHA) hospital, and employees from a university in the Southwestern United States. All participants were aged 35-60 years and were recruited to participate via flyers and targeted emails for a smartphone-based lifestyle behavior program. Inclusion criteria included: (1) insufficient PA (activity ranking category of <4 on the Stanford Leisure-Time Activity Categorical Item) [18], (2) excessive sitting (defined as >8 hours of sitting from the International Physical Activity Questionnaire) [19], (3) short sleep duration (<7 hours/night) or a mild-to-moderate sleep complaint (modified version of the insomnia Severity Index) [20], (4) fasting glucose level of <100 mg/dL, and (5) owning an Android smartphone. All study procedures were approved by the institutional review boards of the local VHA hospital and the university. Each participant underwent telephone screening for eligibility and those who were eligible provided written informed consent.

### BeWell24 App Design

The smartphone app design has been discussed previously [21]. In brief, the smartphone app included four components: (1) a self-monitoring component to allow for rapid self-monitoring of sleep, sedentary time, LPA, and MVPA across the full 24-hour day; (2) a behavioral sleep component that included sleep education, sleep hygiene, and stimulus control therapy; (3) a sedentary component that provided graphical feedback on total sitting time and time spent sitting at work, watching television, socializing, transportation, and other activities; and (4) a PA component that included goal setting, feedback, and problem-solving interventions.

### Study Protocol

Eligible participants were randomized to receive some combination of the sleep, sedentary behavior, and PA components using a full factorial or multiphase optimization strategy [22] study design to optimize efficiency and explore potential synergies among behavioral outcomes (more study design details are provided elsewhere [23]). Briefly, we utilized a full-factorial 2x2x2 screening experiment in which participants were randomized to receive one of eight possible combinations (*k*) of the sleep, sedentary, and exercise components of the app: none (*k*=1), one of three app components (*k*=3), two of three app components (*k*=3), or all three app components (*k*=1). More relevant to the purpose of this study, all participants received access to the self-monitoring component of the app for the entire duration of the study. Using this self-monitoring component, participants were asked to log time spent across 24 hours into domains of sleep, sedentary behavior, LPA, and MVPA throughout the 3-week baseline period and 8-week intervention period. Participants simultaneously wore an activPAL3c accelerometer (criterion) on three 7-day time periods (week 3 as baseline, week 7, and week 11).

### Measures

#### BeWell24 Self-Monitoring App

**Figure 1:**
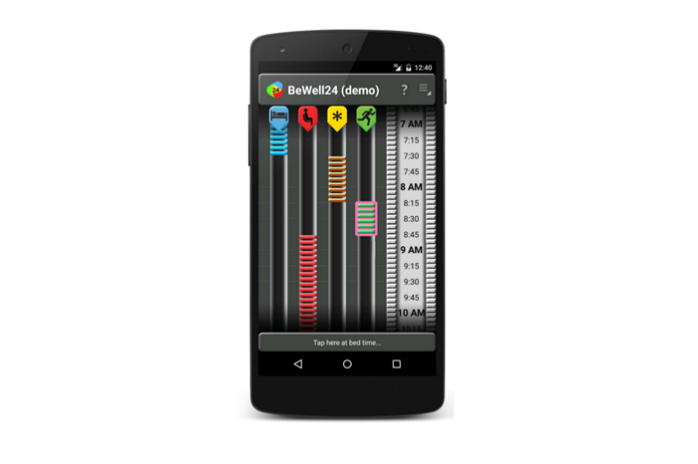
Self-monitoring component of the BeWell24 app.

Self-reported time spent in sedentary behavior, LPA, and MVPA were assessed using the BeWell24 app. The app provided an interface for users to report sleep, sedentary behavior, LPA, and MVPA behaviors in 5-minute epochs across the 24 hours. Figure 1 displays this interface. Users were given standardized definitions of each behavior [21] by tapping the icon on top of each column. Participants were instructed to log any nap or main sleep period, including all time in bed for the purpose of sleep under “Sleep Activities”. Any sitting behaviors (eg, sitting at desk or watching television) were logged in “Sedentary Activities”. All MVPAs such as brisk walking, jogging/walking, and aerobic exercise were logged under “Exercise Activities”. Participants were also instructed to categorize all other activities not fitting into the previous categories (eg, household chores, light gardening, leisurely walking, and other activities of light intensity) under “Other Activities”. For this study’s purposes, all activities categorized under “Other Activities” were classified as LPAs. Users allocated their time into each behavior by dragging their finger down the column throughout the specified time. Although the app was designed to gather contextual information about each of the activities performed, this paper focused on evaluating total time spent on each activity category due to lack of a gold standard measure for these contexts. For the purposes of analyses, time spent in each behavior was summed over all 5-minute epochs after excluding reported sleep time for each day.

#### Criterion Measure of Sedentary and More Active Behaviors

Objective measures were derived from the activPAL3c accelerometer. The devices were waterproofed using medical grade adhesive coverings. Participants wore the device on the midline of their right thigh using breathable hypoallergenic tape, and were instructed to continuously wear the device for 7 days. The validity and reliability of this device in measuring sedentary and PA behaviors have been previously evaluated [24-27]. Collected data were processed into events of sitting, standing, or stepping using the activPAL software version 7.2.32 (PAL Technologies Ltd, Scotland, UK). Self-logged sleep and wake times collected from the BeWell24 app were used to exclude sleep time from the analyses. We used the consensus definition of sedentary behavior as seated/lying positions with low energy expenditure [4]; therefore, all wake time measured by the activPAL3c as lying/seated were considered sedentary. LPA time was defined as time spent standing or stepping at <3 metabolic equivalents (METs). MVPA was defined as time spent stepping at >3 METs. Although the activPAL3c device was primarily developed for measuring sedentary behavior, a recent study has shown that the device is also accurate at classifying and estimating the time spent at these higher-intensity activities (mean bias=-2.6 [-5.8, 0.7] minutes, RMSE=8.4, ICC=0.98 [0.95, 0.99]) [28]. All behaviors were summed to total time spent in that category and expressed in minutes/day. Bouts of continuous sedentary behavior >10 hours from the activPAL3c were considered nonwear time and were excluded from the analyses.

### Statistical Analyses

Data were summarized using means, standard deviations (SDs), frequencies, and percentages. Analyses were performed using SAS Enterprise Guide 6.1 (SAS Institute Inc., Cary, NC) and SPSS version 23 (SPSS, Inc., Chicago, IL).

#### Reliability

Reliability of the BeWell24 app was evaluated using the data collected during the last two weeks of the baseline period. The last two weeks of the baseline period were arbitrarily chosen because they represent a period when participants were well acquainted with the app but before the start of intervention. Days with >80% completion rates on the BeWell24 app were considered valid days, and weeks with less than 3 valid days were excluded from the analyses. The activities spent per day in the second and third baseline weeks were summarized into weekly averages and intraclass correlations (ICCs; 95% CI), with absolute agreement between the two weeks calculated using two-way random effects models [29]. Reliability was considered poor, moderate, or strong when correlation coefficients were <0.4, 0.4-0.8, or >0.8, respectively [21].

#### Validity

**Figure 2:**
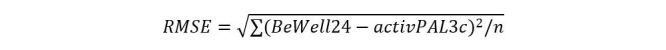
Root mean square error equation.

The validity of the BeWell24 app was evaluated by comparing the app-based reported values to activPAL3c-measured sedentary time, LPA, and MVPA. Days with <80% completion rate of the app or those with <8 hours of valid activPAL3c wear time were excluded from analyses. Agreements between the two measures were assessed using single-measure with absolute definition ICCs using the two-way random effects model. Validity coefficients were interpreted with the scale referenced in the reliability section. Bias estimates such as mean difference (δ) and root of mean square error (RMSE; Figure 2) were also used to determine the degree of over/underestimation of the time spent on each behavior.

The Bland-Altman method was also used to estimate the mean bias and the 95% limits of agreement (2 SD of the difference) between the two measurement methods. The plots were visually inspected for the presence of heteroscedasticity. The degree of heteroscedasticity was then assessed by calculating the Kendall’s tau (τ) correlation between the absolute differences and the corresponding means. When τ>.1, the data were denoted heteroscedastic. When τ<.1 or negative, the data were denoted homoscedastic. If heteroscedasticity was present, the data were transformed by logarithms to the base 10. The limits of agreement from the log transformed data were then transformed back into the original scale by taking antilogs, which were then expressed as a function of the mean in the Bland-Altman plot [22].

#### Responsiveness to Change

**Figure 3:**
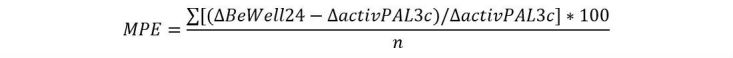


The responsiveness statistic (RS) quantifies the minimal clinically important difference, in relation to the variability in scores of stable participants [30,31]. Ideally, participants are measured multiple times during the baseline and postintervention period to calculate the amount of variability in scores over a stable period. In this analysis, we categorized participants as stable if they did not change their sedentary or LPA time by 30 minutes/day or MVPA by 10 minutes/day. The SDs of the differences in scores between weeks 3 and 7 of these stable participants were used as the denominator in the RS calculation. For each participant, we also used the mean change in time spent in each behavior from week 3 (baseline) to week 7 (∆) as our estimate of the minimal clinically important difference. To supplement our results, we calculated the degree of over/underestimation of the mean change in behavior by the BeWell24 app using mean percentage error (MPE; Figure 3).

#### Data Exclusion

Uncategorized hours in the app and sleep time were excluded from the data analyses. A total of 595 participant days were gathered from participants. Days with <10 hours of activPAL3c wear time and days with >20% annotation (n=219 days, 36.8%) were excluded from the analyses.

## Results

### User Statistics

A total of 26 adults (age 49.0 years, SD 8.9; 85% men; 73% Caucasian; body mass index [BMI]=35.0 kg/m^2^, SD 8.3) participated but only 17 participants completed all aspects of the study. Four subjects withdrew from the study during the 3-week run-in period due to an unrelated health concern (n=1), burdensome assessment protocol (n=2), and loss of contact (n=1). Four subjects were lost to follow-up after randomization (17/21, 81% retention) due to an unrelated health concern (n=1) and loss of contact (n=3). There were no differences in demographic characteristics between *withdrawn* and *lost to follow-up* participants among participants who completed the study (N=17). All available data, from both completers and noncompleters, were included in subsequent analyses. Participants were asked to report their sedentary behavior, LPA, and MVPA in the app over the 11-week study period but only days with matching activPAL3c data (weeks 3, 7, and 11) were included in the analyses. A total of 376 days from 21 participants were analyzed. Across all time points, participants spent an average of 695.5 (SD 139.3) minutes/day being sedentary, 144.9 (SD 112.4) minutes/day on LPAs, and 21.5 (SD 16.5) minutes/day on MVPAs, as measured by the activPAL3c.

### Reliability

Reliability in this study refers to the consistency of the BeWell24 app in measuring a behavior over a stable 2-week period (baseline). Based on the test-retest data, the reliability of the BeWell24 app revealed moderate agreement between measures of total time spent in sedentary behavior, LPAs, and MVPAs (ICC=0.65 [0.43, 0.82], 0.67 [0.44, 0.83], and 0.69 [0.48, 0.84], respectively).

### Validity

**Figure 4:**
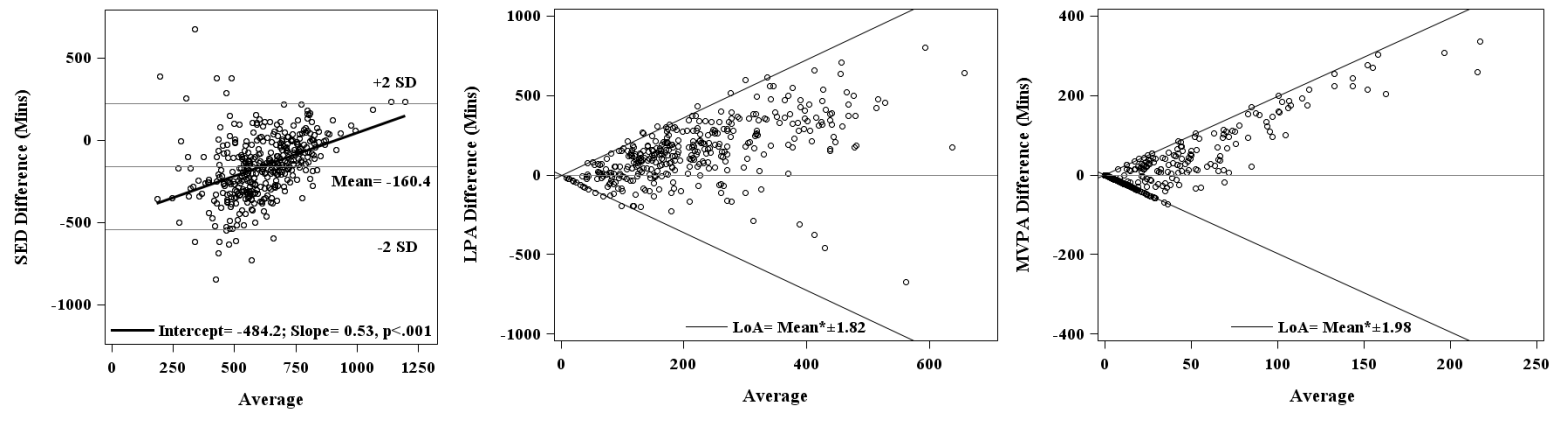
Bland-Altman plot of the BeWell24 app versus activPAL3c. (A) Total time spent sedentary (minutes/day); (B) total time spent in LPAs (minutes/day); (C) total time spent in MVPAs (minutes/day). The y-axes are the difference between the two measures (BeWell24 app – activPAL3c), and the x-axes are the average between the two measures ([BeWell24 app + activPAL3c]/2). SED: sedentary activity; LPA: light-intensity physical activity; MVPA: moderate-vigorous physical activity; LoA: Limits of agreement for heteroscedastic data (calculated as a function of the mean, LoA=±2Mean[10^SD-1]/[10^SD+1]).

Table 1 shows the agreement between the BeWell24 app and activPAL3c in total minutes/day spent in each of the three behaviors. Overall, the agreements between the two measures were poor (ICC range=0.10-0.35). Figure 4 A shows the Bland-Altman plot for self-reported sedentary behavior and activPAL3c-derived total time spent in sedentary activity per day. The data were determined to be homoscedastic (τ=-.35, *P*<.001). Linear regression showed a significant positive bias (β=0.53, *P*<.001) with increasing sedentary time. On average, the BeWell24 app substantially underestimated total sedentary behavior by -160.4 (-179.8, -141.0) minutes/day, and RMSE of 249.5.

**Table 1:** Agreement between activPAL3c and the BeWell24 app.

	Intraclass correlation^a^ (95% CI)	δ^b^ (95% CI)	Root of mean square error
Sedentary Activity	0.35 (0.04, 0.56)	-160.4 (-179.8, -141.0)	249.5
Light-Intensity Physical Activity	0.20 (0.02, 0.36)	144.4 (125.4, 163.5)237.0)	237.0
Moderate-Vigorous Physical Activity	0.10 (-0.01, 0.17)	15.5 (8.8, 22.3)	68.2

^a^Calculated using two-way random effects model with absolute agreement

^b^Mean difference between two measures calculated as δ=Ʃ(BeWell24-activPAL3c)/n

Figure 4B and 4C show the Bland-Altman plot for self-reported and activPAL3c-derived time spent per day in LPA and MVPA activities, respectively. For both LPA and MVPA, visual inspection of the Bland-Altman plots on their original unit of measurement revealed heteroscedasticity, which were confirmed by Kendall’s τ correlation coefficients (0.52 and 0.72, respectively). As such, data were analyzed on the log-transformed scale. There was significant negative bias (β=-0.56, *P*<.001 for LPA, and β=-1.1, *P*<.001 for MVPA). Furthermore, the BeWell24 app significantly overestimated time spent on LPA activities by 144.4 (125.4, 163.5) minutes/day with RMSE of 237.0 and slightly overestimated the time spent on MVPA by 15.5 (8.8, 22.3) minutes/day with RMSE of 68.2.

### Responsiveness to Change

Table 2 depicts the variability of each measure in each activity category in the preintervention period, the mean change from week 3 to week 7, and the RS of the BeWell24 app and activPAL3c. The mean change in sedentary behavior was greater in the BeWell24 app (-17.7 [-91.5, 56.0] minutes/day vs 2.16 [-49.3, 53.6]) minutes/day). However, the RS of the BeWell24 app was lower compared to the activPAL3c (0.20 vs 0.64) due to higher variability in changed scores among stable participants using the app. Similarly, the LPA and MVPA RS of the BeWell24 app was smaller (0.04 vs 2.0, and 0.19 vs 0.40) due to greater variability of changed LPA and MVPA time from the BeWell24 app.

**Table 2:** Responsiveness to change of the BeWell24 app and activPAL3c (n=20).

Variable		% Substantial Changeᵅ (n/N)	Mean T1 and T2 Change (95% CI)	SDᵇ	Responsiveness Statisticsᶜ
**Sedentary Activity**		85 (17/20)			
	BeWell24		-17.7 (-91.5, 56.0)	87.7	0.20
	activPAL3c		2.16 (-49.3, 53.6)	3.4	0.64
**Light-Intensity Physical Activity**		35 (7/20)			
	BeWell24		-3.50 (-44.9, 37.9)	79.7	0.04	
	activPAL3c		-29.2 (-70.4, 11.9)	14.7	2.00
**Moderate-Vigorous Physical Activity**		10 (2/20)			
	BeWell24		-6.40 (-28.8, 15.9)	34.0	0.19
	activPAL3c		-2.00 (-5.00, 0.96)	5.00	0.40

^a^Percentage of participants who decreased by at least 30 minutes/day for sedentary activity, increased by at least 30 mins/day of LPA, or increased 10 minutes/day of MVPA based on activPAL3c

^b^A measure of variability in change score of stable participants

^b^Calculated as (mean change/SD), with direction of change removed

On average, the BeWell24 app slightly underreported change in sedentary behaviors and MVPAs (MPE, 95% CI=-2.0% [-161.7, 157.6.2] and -9% [-316.5, 298.5], respectively). The app significantly overestimated change in LPA time by 207.1% (-28.6 to 442.7). Nevertheless, both measures showed similar directions in change scores for all behaviors.

**Figure 5:**
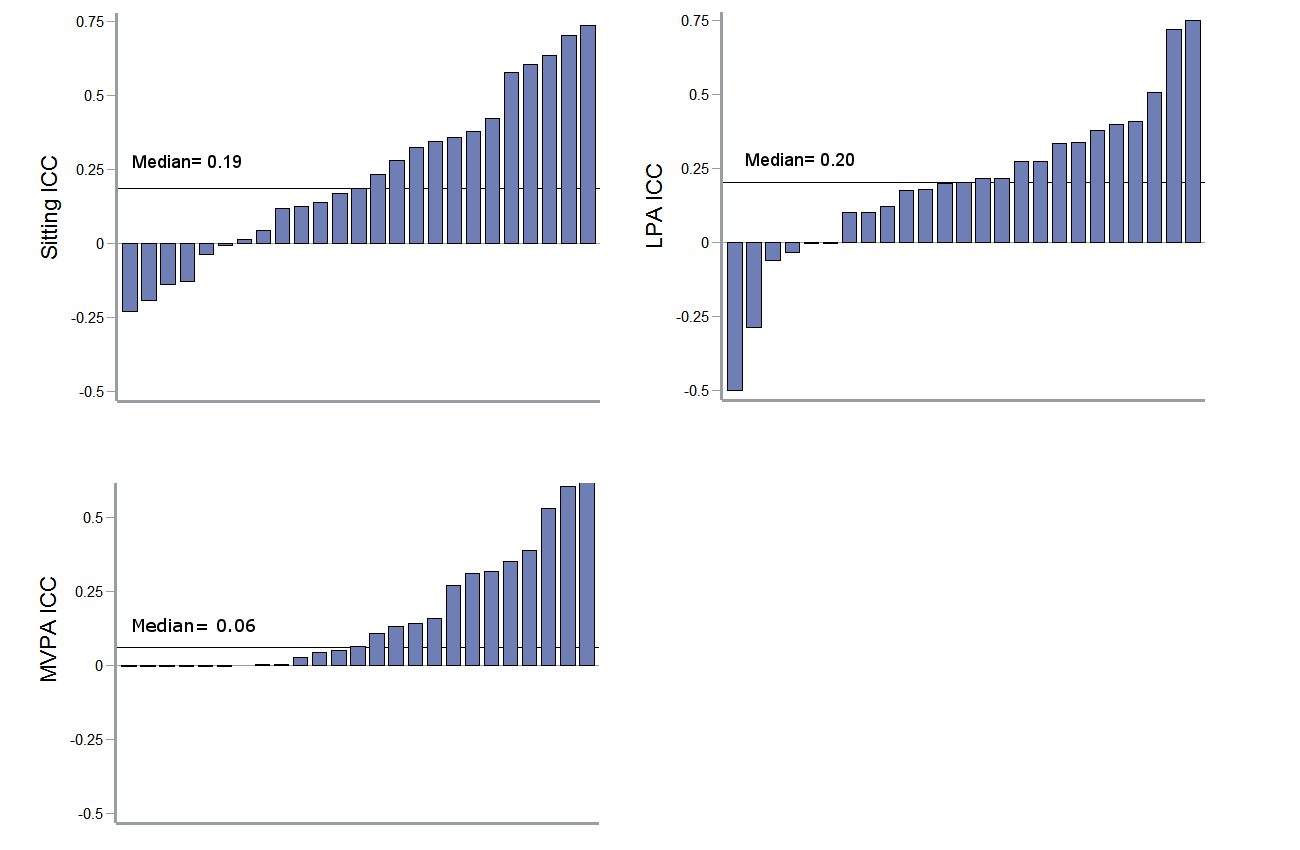
Individual variability in accuracy of self-reported behavior among participants. LPA: light-intensity physical activity; MVPA: moderate-vigorous physical activity; ICC: intraclass correlation.

To understand the reasons for between-subject reporting error, we explored the agreement between the two measures within participants. We observed substantial variation in the agreement of the two measures between subjects (ICC range=-0.19 to 0.74, -0.85 to 0.93, and <-0.001 to 0.75 for sedentary behaviors, LPAs, and MVPAs, respectively; Figure 5). We then evaluated possible predictors of this observed variability using a linear regression model. Multiple variables (age, sex, BMI, activPAL3c-derived total time spent in sedentary behavior, LPA, and MVPA, and total percent of day categorized in the app) were evaluated for their associations with accuracy at reporting sedentary behaviors, LPAs, and MVPAs. We only found a significant positive association in ICC scores between BMI and sedentary behaviors and LPAs (β= 0.026, *P*<0.01 and β=0.025, *P*=.02, respectively), suggesting that participants with higher BMIs tended to be more accurate at reporting their sedentary and LPA behaviors. However, our analysis was limited in power due to a small number of observations within each subject.

**Figure 6:**
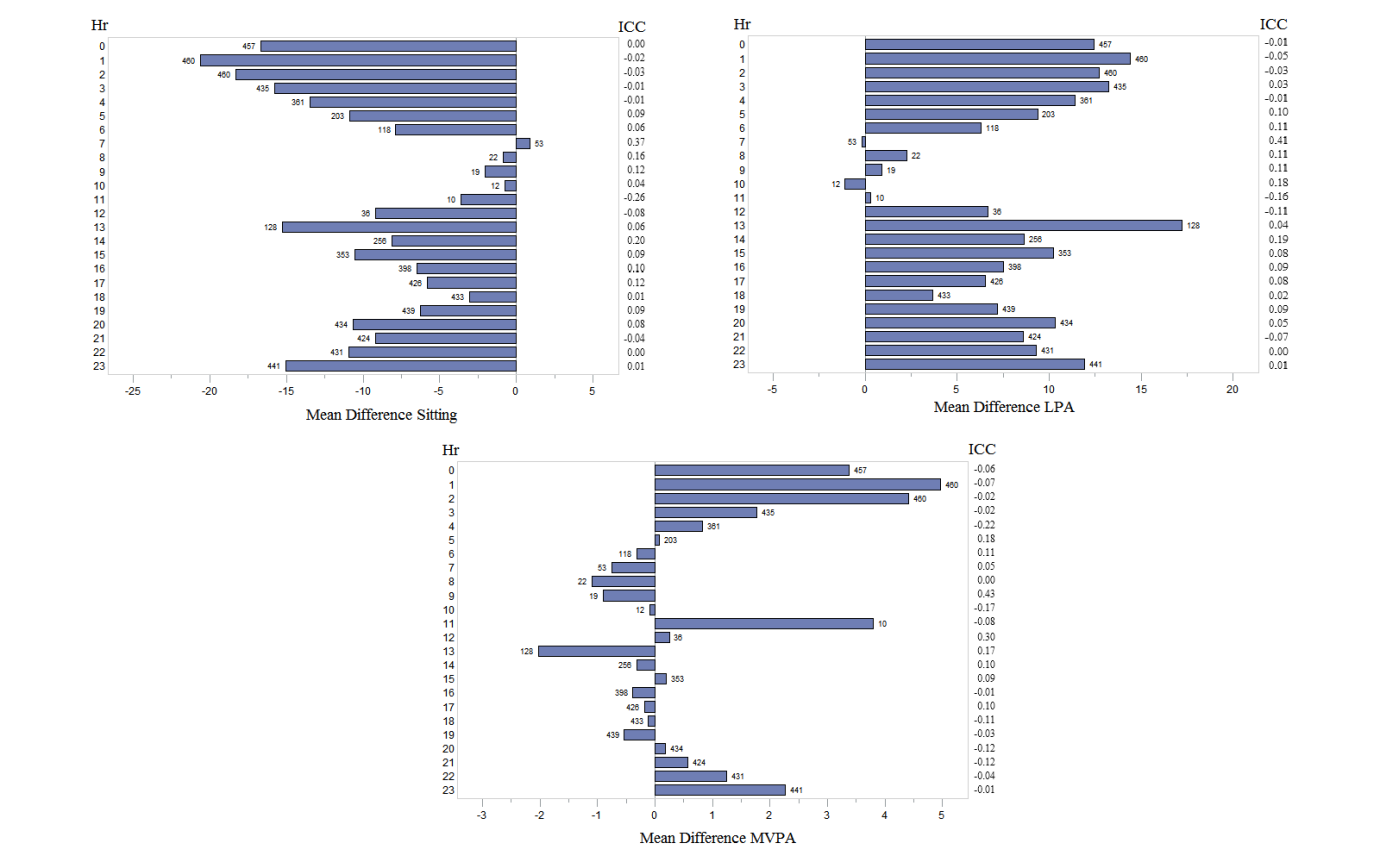
Hourly variability in accuracy of self-reported behavior among participants. The number of observations in each hour is presented beside each bar. Sleep time has been excluded from all analyses. LPA: light-intensity physical activity; MVPA: moderate-vigorous physical activity; ICC: intraclass correlation.

We also explored the accuracy of the reported time spent in each behavior by hour (Figure 6) to understand whether subjects were more accurate in their reporting during morning, midday, or evening times. In general, hourly ICCs ranged from -0.26 to 0.37, -0.16 to 0.41, and -0.22 to 0.43 (for sedentary behaviors, LPAs, and MVPAs, respectively). The overall pattern suggested greater accuracy during morning hours for sedentary and MVPAs, and uniform levels of accuracy for MVPAs during daytime hours with lower accuracy during early morning and late evening hours. The number of observations in each hour are presented beside each bar. Sleep time has been excluded from all analyses.

## Discussion

### Principal Results

This study examined the utility of the BeWell24 app for assessing time spent in sedentary and more active behaviors. The results indicated that the app has moderate reliability and comparable RS to the activPAL3c when measuring sedentary and more active behaviors. This method of sedentary and PA behavior assessment leverages the mobile platform to deliver a more accessible and user-friendly assessment tool. This approach lowers the burden associated with daily diaries and allows for continuous reporting of sleep, sedentary behaviors, LPAs, and MVPAs.

However, our results also indicated poor absolute agreement between the app and the criterion measure for sedentary, LPA, and MVPA behaviors. These results were similar to other paper-based self-report measures of these behaviors (median Spearman of 0.23 for sedentary behavior and 0.30 for total activities) [10]. However, it must be noted that these PA questionnaires were evaluated using Spearman correlations, thus systematic differences between the two measurement methods were not taken into account [32]. Using ICCs to evaluate the reliability and validity of a measure is more suitable because it accounts for these systematic differences, which are considered to be an important element of overall measurement error [33].

Technological advances have led to the development of various mobile technologies that directly measure PA through sensors (ie, accelerometers) integrated into the mobile system. A review that included 10 studies that utilized mobile systems to measure PA found that these systems varied in their ability to classify behaviors (accuracy ranges from 52-100%) [34]. Although these mobile apps do measure behaviors passively and objectively, they are also limited in their ability to detect the context associated with these behaviors. A similar study that used mobile technology to deliver a self-report questionnaire to participants showed that total activity level (as assessed by the questionnaire) was moderately correlated with total counts from the Actigraph (*r*=.45, *P*<.05) [17]. However, the mobile app was only designed to measure total activity level of a person and does not measure the time spent in each activity category. A major advantage of our approach is that we allowed for 24-hour annotation of activity and did not just focus on MVPAs. This factor enabled us to critically evaluate the app for its ability to measure time spent in each activity category. This design will also allow future researchers to evaluate the independent health impacts of these behaviors.

The discrepancies in reported PA behaviors could be due to misclassification of MVPAs to LPAs by the activPAL3c. One limitation of the activPAL3c device, and with other accelerometer-based monitors, is that they are limited in their ability to measure the relative intensity of an activity. For example, a person walking leisurely at 3 miles per hour could be logged as an LPA in the activPAL3c device but could be at moderate intensity relative to the person’s fitness level, which would be reported as MVPA in the app. The under-reporting of LPA time and over-reporting of MVPA time in the BeWell24 app supports this notion. It must also be emphasized that LPA time was calculated from the app using the total time classified as “others”, in which participants were instructed to include behaviors that do not belong to other specified categories. This instruction could have led to some misclassification by the participants. However, participants were given clear and exhaustive instructions when classifying their activities, and any misclassification that occurred could also have randomly occurred in real-life settings, and should be treated as random errors.

Our results also included a responsiveness to change analysis to determine how well the app detects changes in behavior relative to the activPAL3c measure. This change is an important metric to evaluate, given its utility in the context of behavioral interventions, in which the absolute estimate of an activity may be less important than whether change in that activity has occurred. Notably, the app has consistently higher variability in changed scores among stable participants. This result also led the RS scores of the app to be consistently lower compared to the activPAL3c. Regardless, the mean change scores for both the activPAL3c and the BeWell24 app had similar trends in LPA and MVPA categories, suggesting that these measures agree on the overall direction of change for each of the behaviors.

### Strengths and Limitations

One strength of this study is the use of the activPAL3c device, which allowed us to compare the self-report measure to a more valid measure of sedentary behavior and LPA. We also reported the RS of both measures, which enabled us to determine the ability of our instrument to detect changes in our target behavior over time. One key feature of this app that may be useful in future studies is the ability to capture the contextual information of these sedentary and more active behaviors, which greatly improves its utility in interventions targeting domain-specific behaviors. However, this feature was not evaluated in this study due to lack of objective criterion measures. The results from this study will provide useful information for future studies that would evaluate the validity of the app to measure these domain-specific behaviors.

A limitation of our study was the lack of a control group. Due to our study design, all participants received the at least one component of the app (self-monitoring component and/or a combination of the sleep, sedentary, or exercise component). This factor limited our ability to determine whether the observed change in behaviors were due to actual change caused by the intervention, or due to other causes, such as systematic misreporting of the behavior. As pointed out by Gardiner et al [35], a larger RS in our study suggests a greater magnitude of reported change in behavior during the intervention period, and not necessarily a better ability to detect a minimal clinically meaningful change. Furthermore, the activPAL3c device is primarily aimed at measuring sedentary activities and may be limited in its usefulness in measuring MVPAs. The lack of a more accurate measure of active behaviors may have led to the lower validity of the app in measuring MVPA. However, recent studies have shown that although the activPAL3c does overestimate and underestimate the energy expenditure of higher intensity activities, it does a satisfactory job of estimating time spent in these activities [28,36]. We were also limited in our ability to generalize the findings due to our small sample size, which was slightly older and had one or more morbid conditions compared to the general population.

### Conclusions

Our results suggest that the BeWell24 app is reliable and sensitive to change in both sedentary behavior and more active behaviors after an intervention. The analysis showed poor agreement with the activPAL3c when measuring these behaviors. However, this finding is not unexpected, given that using self-reports for absolute measurement of these forms of activities have traditionally been difficult. Despite this limitation, the app is still useful in studies aimed at evaluating interventions targeted at changing these specific behaviors. In addition, the app could be used as a tool to capture context-specific forms of sedentary, LPA, and MVPA behaviors. Further study is needed to evaluate possible correlates for the large amount of between-subject variability in the accuracy of participants when reporting sedentary and more active behaviors.

## Abbreviations

BMI: body mass index
ICC: intraclass correlation
LPA: light-intensity physical activity
MET: metabolic equivalent
MPE: mean percentage error
MVPA: moderate-vigorous physical activity
PA: physical activity
RMSE: root of mean square error
RS: responsiveness statistic
SD: standard deviation
VHA: Veterans Health Administration
δ: mean difference between the app and activPAL3c
∆: mean change from week 3 to week 7
